# Camptothecin, a topoisomerase I inhibitor, impedes productive herpes simplex virus type 1 infection

**DOI:** 10.1128/jvi.01276-25

**Published:** 2025-08-28

**Authors:** Joseph R. Heath, Alison E. Lloyd, Raegen M. Kulinski, Daniel P. Fromuth, Jill A. Dembowski

**Affiliations:** 1 Department of Biological Sciences, Duquesne University 189492, Pittsburgh, Pennsylvania, USA; University of Virginia, Charlottesville, Virginia, USA

**Keywords:** TOP1, beta-lapachone, camptothecin, herpes simplex virus, HSV-1, topoisomerase I, DNA topology, transcription, DNA replication

## Abstract

Herpes simplex virus type 1 (HSV-1) is a double-stranded DNA virus that infects most of the human population. We previously found that cellular topoisomerase I (TOP1) associates with the HSV-1 genome throughout infection. TOP1 relieves topological stress on DNA to enable and regulate transcription, DNA replication, and DNA repair. We hypothesized that TOP1 contributes to these key viral processes because HSV-1 does not encode a topoisomerase. We found that TOP1 inhibition with camptothecin (CPT) or β-Lapachone results in a significant reduction in viral yield. The effects of CPT are reversible and occur in a dose-dependent manner, independent of strain and cell type. CPT treatment results in a decrease in viral gene expression and DNA replication, with replication defects observed at all concentrations tested. Defects in viral gene expression are not restricted to a specific gene class. However, the timing and extent of the observed defects are dose dependent. We also demonstrate that if CPT is added to cells after the onset of viral DNA replication, subsequent rounds of replication are inhibited, indicating that the replication defect observed is not simply the result of prior steps in the infectious cycle being blocked. Together, these results demonstrate that CPT is a potent inhibitor of HSV-1 infection, supporting the hypothesis that TOP1 is active on HSV-1 genomes during infection.

**IMPORTANCE:**

HSV-1 is a common human pathogen. It utilizes both viral and cellular factors to facilitate infection. We previously characterized the proteins that associate with HSV-1 DNA throughout infection. One of these proteins, which has been largely unexplored in the context of HSV-1 infection, is TOP1. TOP1 functions to relieve topological stress to regulate transcription, DNA replication, and other processes that involve DNA cleavage and unwinding. Our findings support a role for TOP1 in HSV-1 DNA replication and gene expression and highlight the potential to target TOP1 activity or interactions for antiviral therapy.

## INTRODUCTION

HSV-1 is a double-stranded DNA virus that infects over 67% of the adult population (1). HSV-1 infection can result in a variety of clinical manifestations, including cold sores, keratitis, and encephalitis (2–5). Primary infection occurs in epithelial cells, followed by the establishment of latency in peripheral neurons. During productive infection, HSV-1 undergoes a highly ordered temporal cascade of gene expression (6–12). Upon entry of the viral genome into the nucleus, viral factors initiate the expression of immediate early (IE) genes. IE gene products then facilitate the expression of early (E) genes and the onset of leaky late (LL) gene expression. E gene products are essential for viral DNA replication, which results in an increase in LL and the onset of late (L) gene expression. L genes encode structural and packaging proteins for virion assembly. As the HSV-1 genome does not encode an RNA polymerase, cellular RNA polymerase II (Pol II) transcribes all viral genes (13, 14).

Topological stress occurs when DNA unwinds, which results in the formation of supercoils (15). During transcription, overwound DNA ahead of the transcription bubble generates positive supercoils, whereas underwound DNA behind the transcription bubble generates negative supercoils (16). Similarly, during DNA replication, positive supercoils form ahead of the replication fork (17). Supercoiling regulates the synthesis of nascent RNA and DNA, as well as chromatin structure and nuclear architecture (15, 17–19).

Type I and II topoisomerases regulate DNA topology by causing transient single or double-strand breaks, respectively (20). TOP1 is a type I topoisomerase that functions in transcription and chromatin organization, as well as DNA replication, repair, and recombination (20–26). The TOP1 active site tyrosine catalyzes strand cleavage by nucleophilic attack of the DNA backbone, forming a covalent DNA 3´-phosphotyrosyl linkage called the TOP1-DNA covalent cleavage complex (TOP1cc) (27, 28). The nicked strand then rotates around the intact strand and is re-ligated, resulting in relief of topological stress.

**Fig 1:**
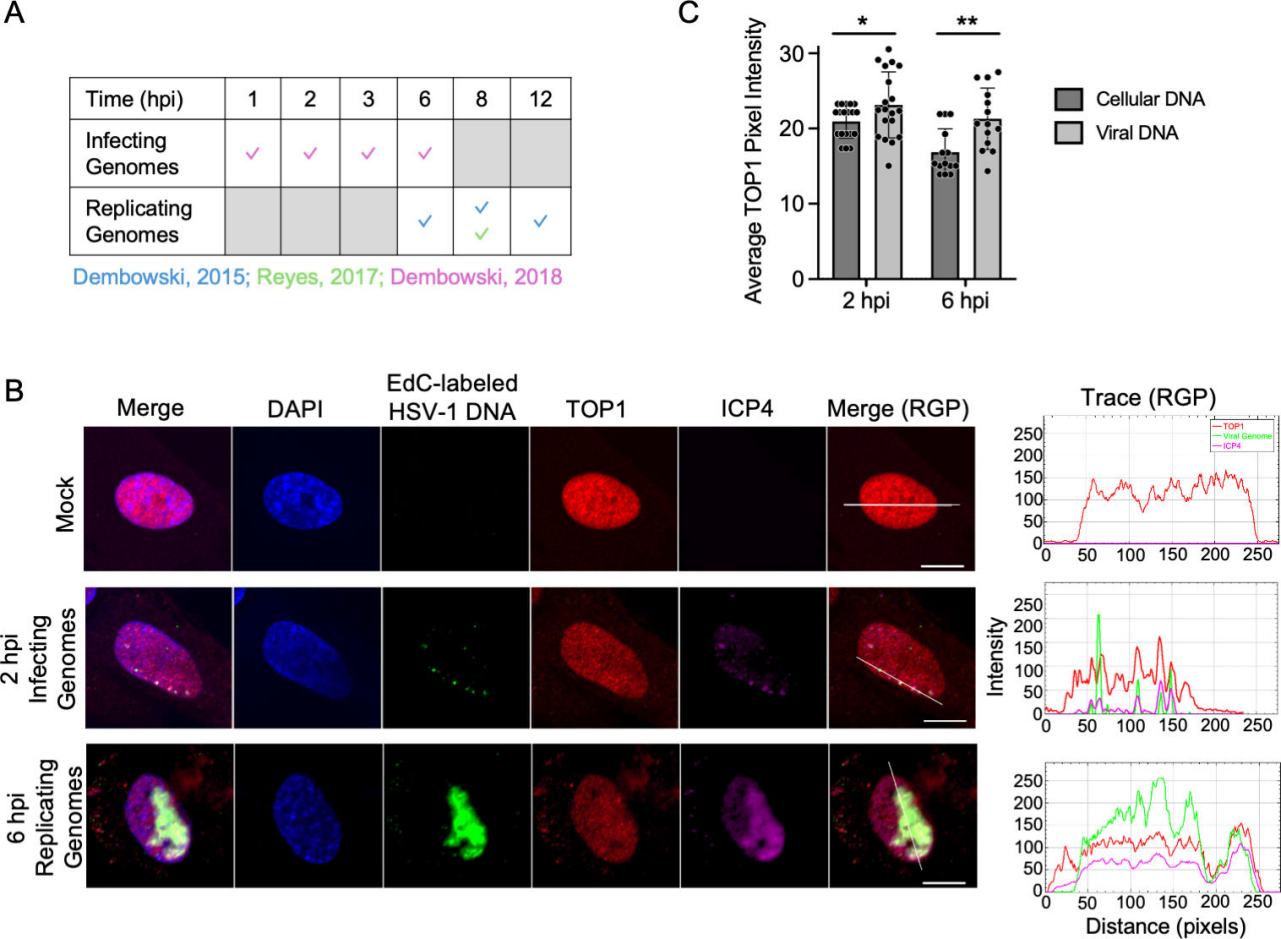
TOP1 colocalizes with HSV-1 DNA. (**A**) TOP1 copurifies with both infecting and replicating HSV-1 genomes by the isolation of proteins on nascent DNA (iPOND) or accelerated native iPOND (aniPOND). Checks indicate TOP1 copurification with viral DNA at that time point/condition, and gray boxes indicate that that time point/condition was not tested. (**B**) TOP1 colocalizes with HSV-1 DNA. MRC-5 cells were mock-infected (Mock), infected with EdC-labeled HSV-1 strain KOS (2 hpi infecting genomes), or infected with KOS (6 hpi replicating genomes) at an MOI of 10 PFU/cell. Cells were fixed at either 2 hpi, or replicating viral DNA was labeled with EdC from 4 to 6 hpi, and then fixed at 6 hpi. Cellular DNA was stained with DAPI (blue), EdC-labeled viral DNA was tagged with Alexa Fluor 488 using click chemistry (green), and TOP1 (red) and ICP4 (purple) were imaged by indirect immunofluorescence. Merge displays all channels for each cell, and Merge (RGP) is the same, with DAPI omitted. Scale bars represent 10 µm. Trace files, generated in ImageJ, show the colocalization of viral DNA, TOP1, and ICP4 along the line in the Merge (RGP) panel. (**C**) TOP1 pixel intensity is elevated in regions of viral DNA compared with cellular DNA. Average TOP1 pixel intensity was determined within regions of viral and cellular DNA using the ROI Manager in ImageJ. Data were generated from multiple images. Error bars represent standard deviations, and significance was determined by paired *t*-tests with pixel intensity of viral and cellular DNA compared within individual cells. *P*-values: * ≤0.05, ** ≤0.01, *** ≤0.001.

Multiple studies have demonstrated that TOP1 associates with HSV-1 DNA throughout infection, as summarized in Fig. 1A (29–31). TOP1 colocalizes with HSV-1 replication compartments (30, 32) and copurifies with infecting viral DNA from 1 to 6 hpi (31) and replicated viral DNA from 6 to 12 hpi (29, 30). Inhibition of TOP1 with CPT, a chemotherapeutic drug that reversibly captures TOP1 in a covalent complex with DNA (33), results in a reduction in herpes simplex virus type-2 (HSV-2) gene expression (34). Because HSV-2 and HSV-1 are closely related, sharing approximately 50% sequence homology (35), TOP1 may also contribute to HSV-1 infection.

In this study, we used CPT to inhibit TOP1 during HSV-1 infection. CPT was first characterized as a chemotherapeutic agent (33), with TOP1 later being identified as its specific target (36). When TOP1 is either knocked out or mutated, the cells exhibit resistance to the effects of CPT (37–39). Additionally, several CPT derivatives have been developed as cancer treatments. Collectively, these findings establish CPT as a well-characterized inhibitor of TOP1 and support its use in this study.

We investigated the effects of CPT inhibition of TOP1 on HSV-1 infection and observed a significant reduction in viral yield. To identify the steps in the infectious cycle that involve TOP1 activity, we monitored viral gene expression and DNA replication during the infection of CPT-treated cells. We found that TOP1 inhibition results in dose-dependent defects in viral gene expression and DNA replication, even when CPT is added following the onset of viral DNA replication. This indicates that defects in later stages of infection are not only the result of IE and E gene expression defects that occur when added before infection. These results reveal that CPT inhibits HSV-1 infection, suggesting that TOP1 is active on the HSV-1 genome throughout infection and contributes to viral transcription and DNA replication.

## RESULTS

### TOP1 colocalizes with HSV-1 genomes throughout infection

To investigate the role of TOP1 in HSV-1 infection, we initially confirmed the colocalization of TOP1 with HSV-1 DNA (Fig. 1B). MRC-5 cells were mock-infected or infected with either HSV-1 strain KOS containing 5-ethynyl-2´-deoxycytidine (EdC)-labeled DNA (KOS-EdC) to investigate infecting viral genomes or KOS to investigate replicating genomes. Cells were either fixed at 2 hpi or replicating viral DNA was labeled with EdC from 4 to 6 hpi and then fixed at 6 hpi. EdC-labeled viral DNA was covalently attached to Alexa Fluor 488 (green), nuclei were stained with DAPI (blue), and TOP1 (red) and infected cell protein 4 (ICP4) (purple) were visualized by indirect immunofluorescence. ICP4 is the major viral transcription factor that is expressed in infected cells before 2 hpi and binds to double-stranded viral DNA (40). As expected, neither EdC-labeled viral DNA nor ICP4 was detected in uninfected cells. In infected cells at 2 and 6 hpi, TOP1 colocalizes with infecting viral genomes (2 hpi) and viral replication compartments (6 hpi), as well as ICP4, as indicated in the trace images at right. Although TOP1 does not exclusively localize to viral genomes, there is an enrichment of TOP1 at the sites of viral DNA compared with cellular DNA (Fig. 1C). These results support previous observations that TOP1 associates with viral genomes at both early and late times during productive HSV-1 infection (29–32, 41).

### CPT treatment results in reduced viral yield

**Fig 2:**
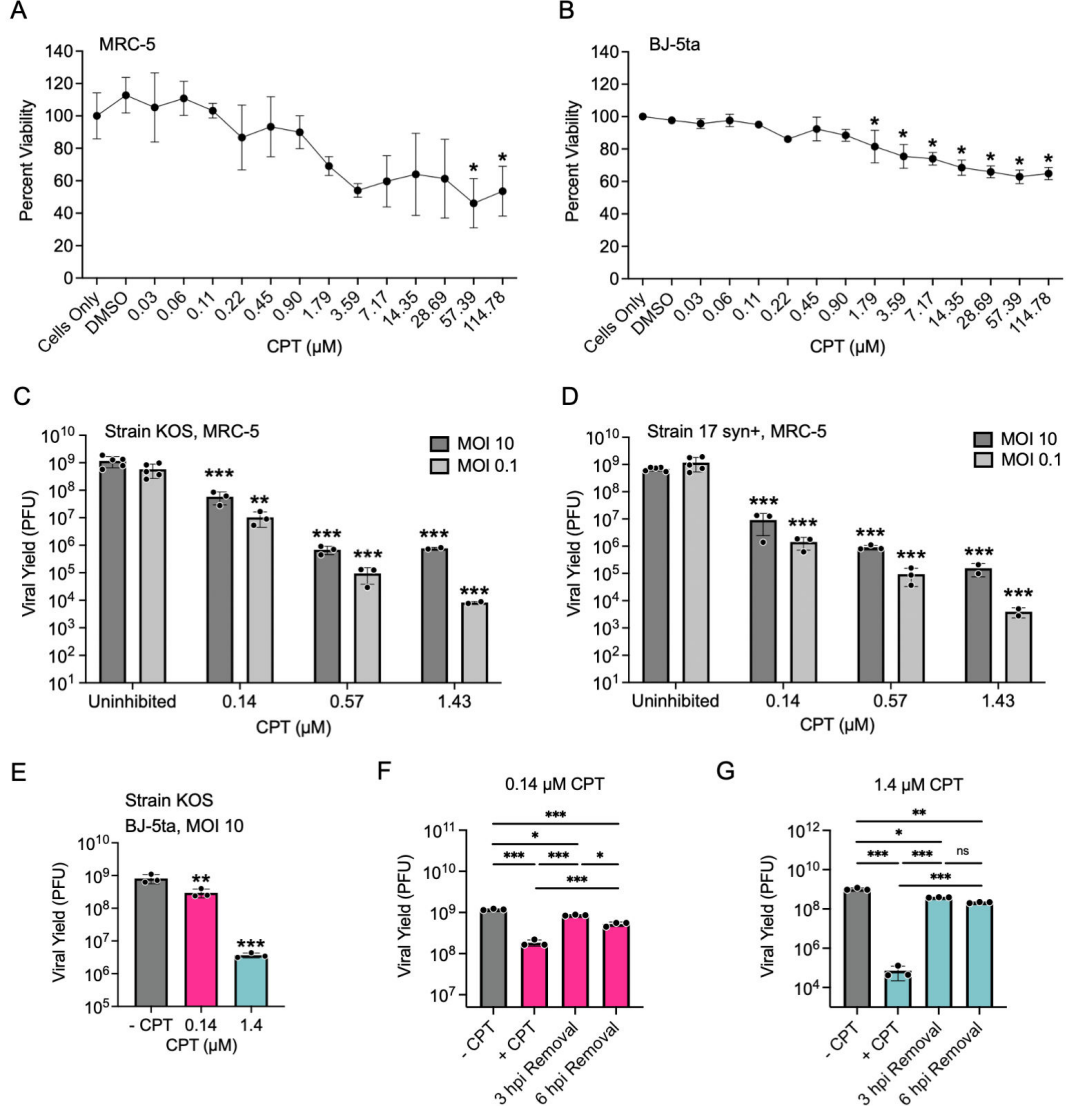
CPT treatment results in a reversible, dose-dependent, strain-independent decrease in viral yield at noncytotoxic concentrations. (**A-B**) Cytotoxicity of CPT in MRC-5 and BJ-5ta cells. Using the CellTiter-Glo Luminescence Cell Viability Assay, the viability of either MRC-5 (**A**) or BJ-5ta (**B**) cells was determined after growth for 24 h upon treatment with increasing concentrations of CPT. Error bars represent standard deviations. *P-value ≤ 0.01 as determined by one-way ANOVA followed by Dunnett’s multiple comparison test when compared with the “cells-only” control. (**C-D**) CPT treatment results in a dose- and MOI-dependent decrease in viral yield. MRC-5 cells were treated with increasing concentrations of CPT and infected with HSV-1 strain KOS (**C**) or 17 syn+ (**D**) at either an MOI of 10 or 0.1 PFU/cell. Virus was collected at 24 hpi and titered in Vero cells. Error bars represent standard deviations of 3 biological replicates. Statistical analyses were performed on log10-transformed data to compare each CPT-treated group with the corresponding uninhibited control. *P*-values: * ≤0.05, ** ≤0.01, *** ≤0.001, as determined by one-way ANOVA followed by Dunnett’s multiple comparison test. (**E**) CPT treatment results in reduced viral yield in BJ-5ta cells. BJ-5ta cells were treated with 0, 0.14, or 1.4 µM CPT, then infected with HSV-1 at an MOI of 10 PFU/cell. At 24 hpi, the virus was collected and titered in Vero cells. Error bars represent the standard deviation of 3 biological replicates. Statistical analyses were performed on log10-transformed data to compare each CPT-treated group with the corresponding uninhibited control. *P*-values: * ≤0.05, ** ≤0.01, *** ≤0.001 as determined by one-way ANOVA followed by Dunnett’s multiple comparison test. (**F-G**) CPT treatment is reversible, and removal partially restores viral yield. MRC-5 cells were infected at an MOI of 10 PFU/cell and subject to no CPT inhibition (-CPT) or treated with 0.14 (**F**) or 1.4 (**G**) µM CPT. During infection, CPT-containing medium was either not removed (+CPT) or removed and replaced with CPT-free medium at 3 hpi (3 hpi removal) or 6 hpi (6 hpi removal). At 24 hpi, the virus was collected and titered in Vero cells. Error bars represent the standard deviation of 3 biological replicates. Statistical analyses were performed on log10-transformed data to compare each CPT-treated group with the corresponding uninhibited control. *P*-values: * ≤0.05, ** ≤0.01, *** ≤0.001 as determined by one-way ANOVA, followed by Dunnett’s multiple comparison test.

To understand the potential role of TOP1 in HSV-1 infection, we determined the effect of CPT-mediated inhibition of TOP1 on viral yield. First, we conducted a CellTiter-Glo Luminescent Cell Viability Assay to identify concentrations of CPT that do not significantly affect cell viability. This assay measures adenosine triphosphate (ATP) levels as a proxy for cell viability. We tested the effects of CPT treatment on two human diploid fibroblast cell lines, MRC-5 (Fig. 2A) and BJ-5ta (Fig. 2B). For MRC-5 cells, there was a statistically significant reduction in cell viability at 57.39 and 114.78 µM CPT. For BJ-5ta cells, there was a statistically significant reduction in cell viability at greater than 1.79 µM CPT. The variance in cell viability for MRC-5 cells may mask significance between 1.79 and 28.69 µM CPT when compared to the uninhibited control, as seen in BJ-5ta cells. As a result, we chose to proceed using concentrations below this range, specifically 0.14 and 1.4 µM CPT. Note that there is a high correlation between CPT sensitivity and cell growth rates, with slower-growing cells able to tolerate higher doses of CPT (42). Therefore, for all subsequent experiments, MRC-5 or BJ-5ta cells were grown to confluency to enable contact inhibition before the addition of CPT and infection.

Next, we determined how CPT treatment affects viral yield. MRC-5 cells were infected with either HSV-1 strain KOS (Fig. 2C) or strain 17 syn+ (Fig. 2D) at a multiplicity of infection (MOI) of 0.1 or 10 plaque-forming units (PFU)/cell. CPT was maintained in the growth medium throughout infection, and viral yield was determined after 24 h. CPT treatment results in a dose-dependent, but strain-independent decrease in viral yield at both low and high MOI. In addition, CPT treatment results in a dose-dependent reduction in viral yield when BJ-5ta cells were infected with KOS when treated with 0.14 or 1.4 µM CPT (Fig. 2E). Therefore, the inhibitory effect of CPT on infection is not cell type-specific. CPT stops re-ligation after cleavage and formation of TOP1ccs, resulting in prolonged TOP1 covalent attachment to DNA and a single-stranded nick in the DNA. Thus, inhibition of viral infection by CPT is consistent with TOP1 catalytic activity playing a role in HSV-1 infection. For all subsequent experiments, strain KOS was used for infections, which were carried out at an MOI of 10 in MRC-5 cells treated with 0, 0.14, or 1.4 µM CPT.

### CPT inhibition of viral infection is reversible

CPT is a reversible inhibitor of TOP1. To assess this property during viral infection, MRC-5 cells were infected at an MOI of 10 PFU/cell and subjected to treatment with no CPT, 0.14 (Fig. 2F), or 1.4 (Fig. 2G) µM CPT. During infection, CPT-containing medium was either not removed (+CPT) or was removed and replaced with CPT-free medium at either 3 (3 hpi removal) or 6 (6 hpi removal) hpi. At 24 hpi, the virus was harvested and titered by plaque assay. Consistent with Fig. 2C, CPT treatment resulted in a dose-dependent reduction in viral yield. When CPT was removed at 3 or 6 hpi, there was an increase in yield compared with the +CPT group. Removal of CPT at 6 hpi resulted in a smaller increase in yield than removal at 3 hpi. Regardless of the time of removal, the titers were restored to levels comparable with the uninhibited control. These data demonstrate that CPT treatment under these conditions did not trigger an irreversible DNA damage response or cell death, thus supporting a direct effect of CPT on infection.

### CPT treatment results in a reduction in HSV-1 yield regardless of the time of addition

**Fig 3:**
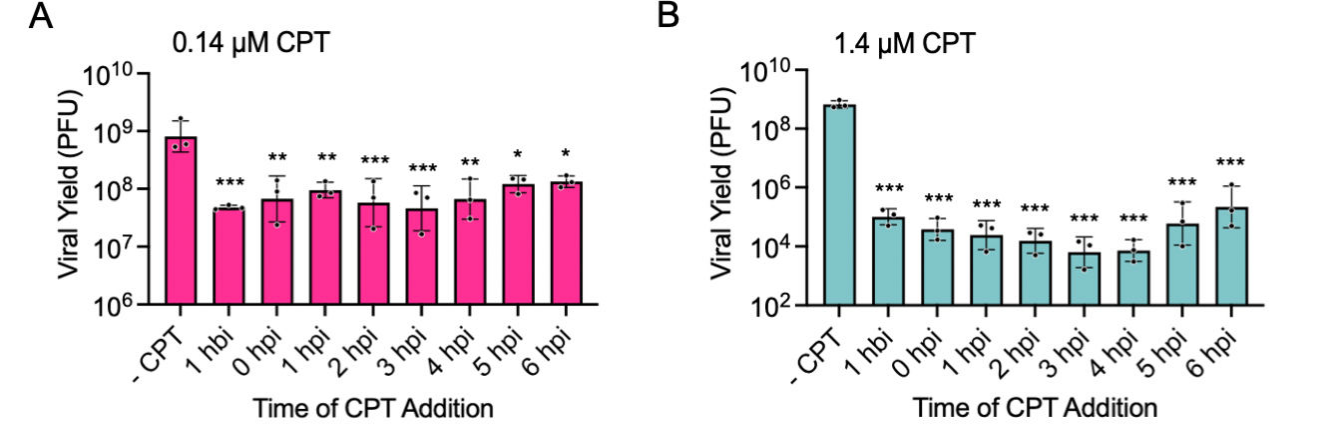
HSV-1 infection is inhibited when CPT is added after the onset of viral gene expression and DNA replication. (**A-B**) MRC-5 cells were infected with KOS at an MOI of 10 PFU/cell, and either 0.14 µM (**A**) or 1.4 µM (**B**) CPT was added at the indicated times. At 24 hpi, the virus was harvested and titered by plaque assay. Error bars represent standard deviations of three biological replicates. Statistical analyses were performed on log10-transformed data to compare each CPT-treated group with the corresponding uninhibited control. *P*-values: * ≤0.05, ** ≤0.01, *** ≤0.001, as determined by one-way ANOVA, followed by Dunnett’s multiple comparison test.

To assess whether CPT inhibits early or late stages of infection, we quantified viral yield when CPT (either 0.14 or 1.4 µM) was added at various times before or after infection, followed by the collection of virus at 24 hpi (Fig. 3). There was a statistically significant reduction in viral yield regardless of the time of addition, suggesting that the effects of CPT on viral yield are not a result of inhibition of early steps in infection such as viral entry into cells, intracellular trafficking, or nuclear entry. Furthermore, CPT could still inhibit infection if added after the onset of viral DNA replication (4–6 hpi), although the effects are slightly reduced. Taken together, these data suggest that CPT inhibits ongoing viral gene expression, DNA replication, or later stages of infection because inhibition of early stages of infection is not required for a reduction in viral yield.

### CPT acts through TOP1 inhibition to restrict viral infection

**Fig 4:**
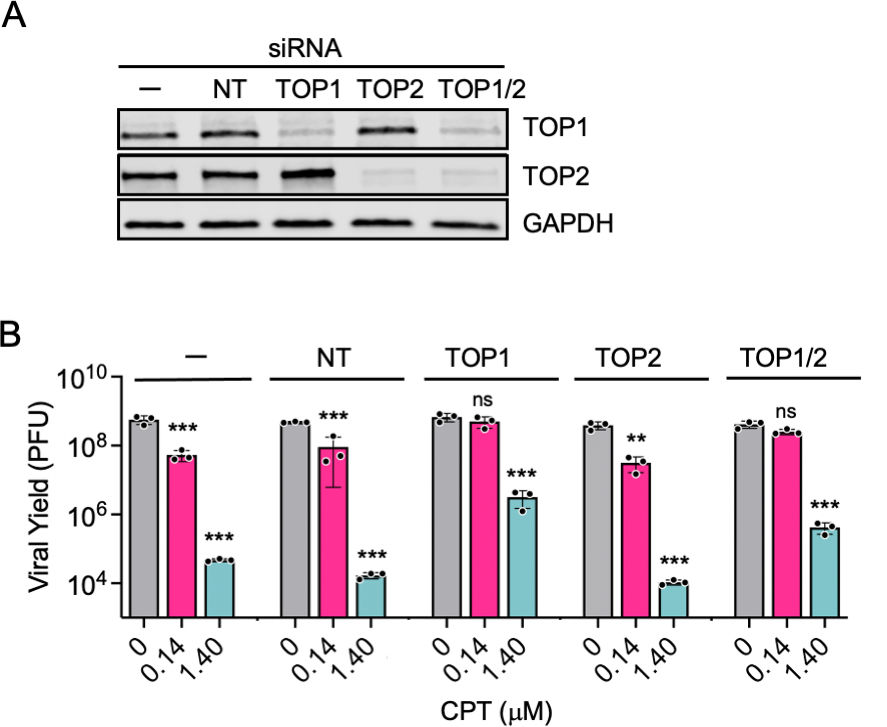
CPT targets TOP1 to inhibit HSV-1 infection. (**A**) Western blotting to confirm siRNA knockdown. MRC-5 cells were transfected with TOP1, TOP2 (TOP2A and TOP2B), or TOP1 and TOP2 (TOP1/2) targeting siRNAs or a non-targeting (NT) control. After 48 h, proteins were collected and subjected to western blotting with antibodies specific for TOP1, TOP2, or glyceraldehyde-3-phosphate dehydrogenase (GAPDH) as a loading control. (**B**) TOP1 knockdown reduces the effects of CPT treatment on viral yield. Knockdown was carried out as described in (**A**), and then, the cells were treated with 0, 0.14, or 1.4 µM CPT, followed by infection with HSV-1 strain KOS at an MOI of 10 PFU/cell. After 24 h, the viral yield was determined by plaque assay. *P*-values: * ≤0.05, ** ≤0.01, *** ≤0.001 were determined by one-way ANOVA, followed by Tukey’s multiple comparison test. Significance is shown to compare each treated group with the corresponding untreated control. There was no significant difference between any of the untreated groups (gray bars).

We next carried out siRNA knockdown of TOP1, as well as topoisomerase 2 (TOP2), to assess whether depletion of either topoisomerase mimics the antiviral effects of CPT or alters the compound’s efficacy in suppressing viral infection. For TOP2 knockdown, siRNAs that target both TOP2A and TOP2B were combined and added to cells. Knockdown was verified by western blotting (Fig. 4A), and the effects on viral yield were determined by plaque assay (Fig. 4B). In the absence of CPT (gray bars), knockdown of TOP1, TOP2, or topoisomerases in combination (TOP1/2) had no effect on viral yield, indicating that these enzymes are not essential for high MOI infection. After treatment with no siRNA(−), a non-targeting (NT) siRNA control, or a pool of siRNAs that target TOP2, CPT treatment resulted in a dose-dependent decrease in viral yield as observed in Fig. 2B. For the NT control, treatment with 0.14 µM CPT resulted in ~5-fold reduction in viral yield and treatment with 1.4 µM CPT resulted in ~10,000 fold reduction. However, if CPT treatment and infection occur after TOP1 knockdown alone or in combination with TOP2 knockdown, 0.14 µM CPT did not affect viral yield, and the effects of 1.4 µM CPT were significantly reduced, resulting in ~200-fold reduction in yield. Taken together, these data demonstrate that CPT acts through TOP1 inhibition to restrict viral infection, and at higher concentrations, there may be non-specific effects of CPT on viral yield.

### β-Lapachone inhibits HSV-1 infection

**Fig 5:**
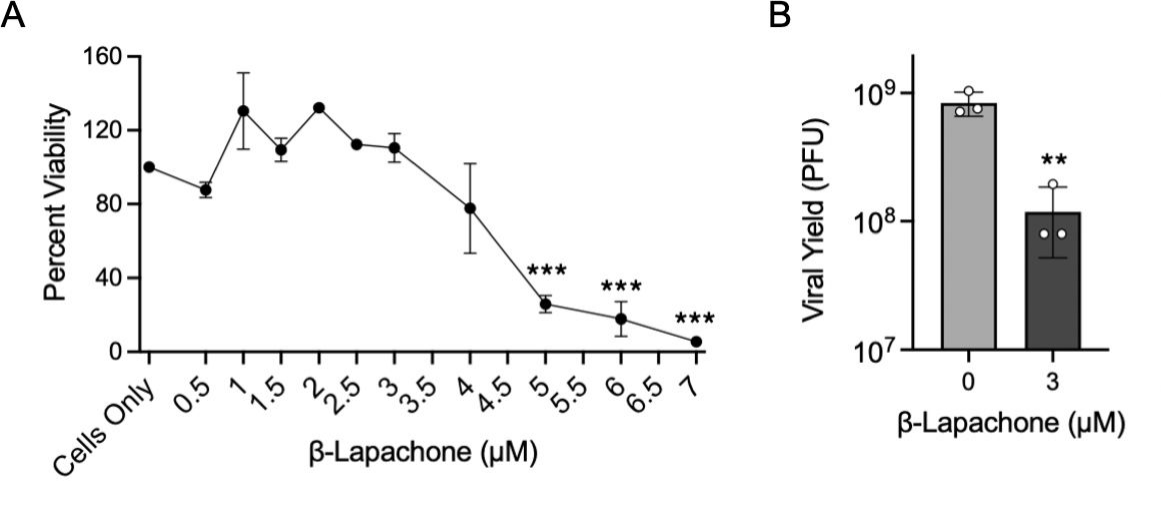
β-Lapachone treatment results in a decrease in viral yield at noncytotoxic concentrations. (**A**) Cytotoxicity of β-Lapachone in MRC-5 cells. Using the CellTiter-Glo Luminescence Cell Viability Assay, the viability of MRC-5 cells was determined after growth for 24 h in the presence of increasing concentrations of β-Lapachone. Error bars represent standard deviation. *P*-values: * ≤0.05, ** ≤0.01, *** ≤0.001 as determined by one-way ANOVA followed by Dunnett’s multiple comparison test when compared with the “cells-only” control. (**B**) β-Lapachone treatment results in reduced viral yield. MRC-5 cells were treated with 0 or 3 µM β-Lapachone and infected with HSV-1 strain KOS at an MOI of 10 PFU/cell. The virus was collected after 24 h and titered in Vero cells. Error bars represent standard deviations of three biological replicates. Significance was determined by an unpaired *t*-test with *P*-values: * ≤0.05, ** ≤0.01, *** ≤0.001.

To determine if the effects of CPT on viral yield relate to the formation of TOP1ccs on the DNA, we tested the effects of another TOP1 inhibitor, β-Lapachone, on viral yield. β-Lapachone is a plant product that inhibits the catalytic activity of TOP1, but unlike CPT, it does not stabilize the covalent complex between TOP1 and DNA (43). We first carried out a CellTiter-Glo Luminescent Cell Viability Assay to identify the concentrations of β-Lapachone that do not significantly affect MRC-5 cell viability (Fig. 5A). There was a statistically significant reduction in cell viability at and above 4 µM β-Lapachone, consistent with published data. We next determined how treatment with β-Lapachone affects viral yield (Fig. 5B). MRC-5 cells were treated with 0 or 3 µM β-Lapachone for 1 h before and during infection with HSV-1 strain KOS, and the viral yield was determined after 24 h. β-Lapachone treatment resulted in a 7-fold reduction in viral yield, having a similar effect as 0.14 µM CPT treatment. These data indicate that although TOP1 is not essential for viral infection, inhibition of TOP1 activity during infection is detrimental to the HSV-1 life cycle.

### TOP1 inhibition with CPT results in a viral DNA replication defect

**Fig 6:**
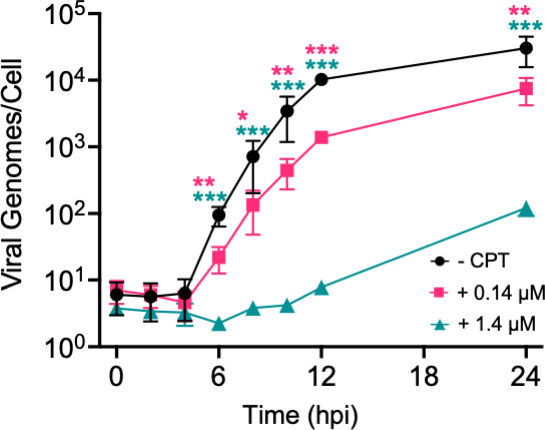
CPT inhibits viral DNA replication. MRC-5 cells were infected at an MOI of 10 PFU/cell in the absence or presence of 0.14 or 1.4 µM CPT. Total DNA was isolated at 2-h intervals from 0 to 12 hpi, and at 24 hpi. DNA was subject to qPCR using primers that recognize the viral thymidine kinase (TK) and cellular GAPDH genes. Genome quantity was determined relative to standard curves of known quantities of viral or human DNA. The number of human genomes was used to determine the number of cells isolated. Each data point represents the mean of three biological replicates. Error bars represent the standard deviation from the mean. *P*-values: * ≤0.05, ** ≤0.01, *** ≤0.001 as determined by one-way ANOVA followed by Dunnett’s multiple comparison test comparing +0.14 (pink) or +1.4 µM (teal) CPT conditions to the uninhibited control. Note that the absence of an asterisk indicates no statistical significance. Statistical analyses for these data sets were carried out on log10-transformed data.

To examine how TOP1 inhibition affects HSV-1 DNA replication, MRC-5 cells were infected and treated with no CPT, 0.14, or 1.4 µM CPT, and total DNA was isolated every 2 h from 0 to 12 hpi and at 24 hpi. Quantitative real-time PCR (qPCR) was used to determine the number of viral genomes produced per cell during infection (Fig. 6). In samples collected after the onset of viral DNA replication (after 4 hpi), there was a decrease in the number of viral genomes per cell at either concentration of CPT, with a more exaggerated effect at 1.4 µM CPT. These results indicate that CPT treatment results in a dose-dependent defect in HSV-1 DNA replication.

### CPT inhibition increases TOP1 colocalization with HSV-1 genomes and results in a reduction in replication compartment size

**Fig 7:**
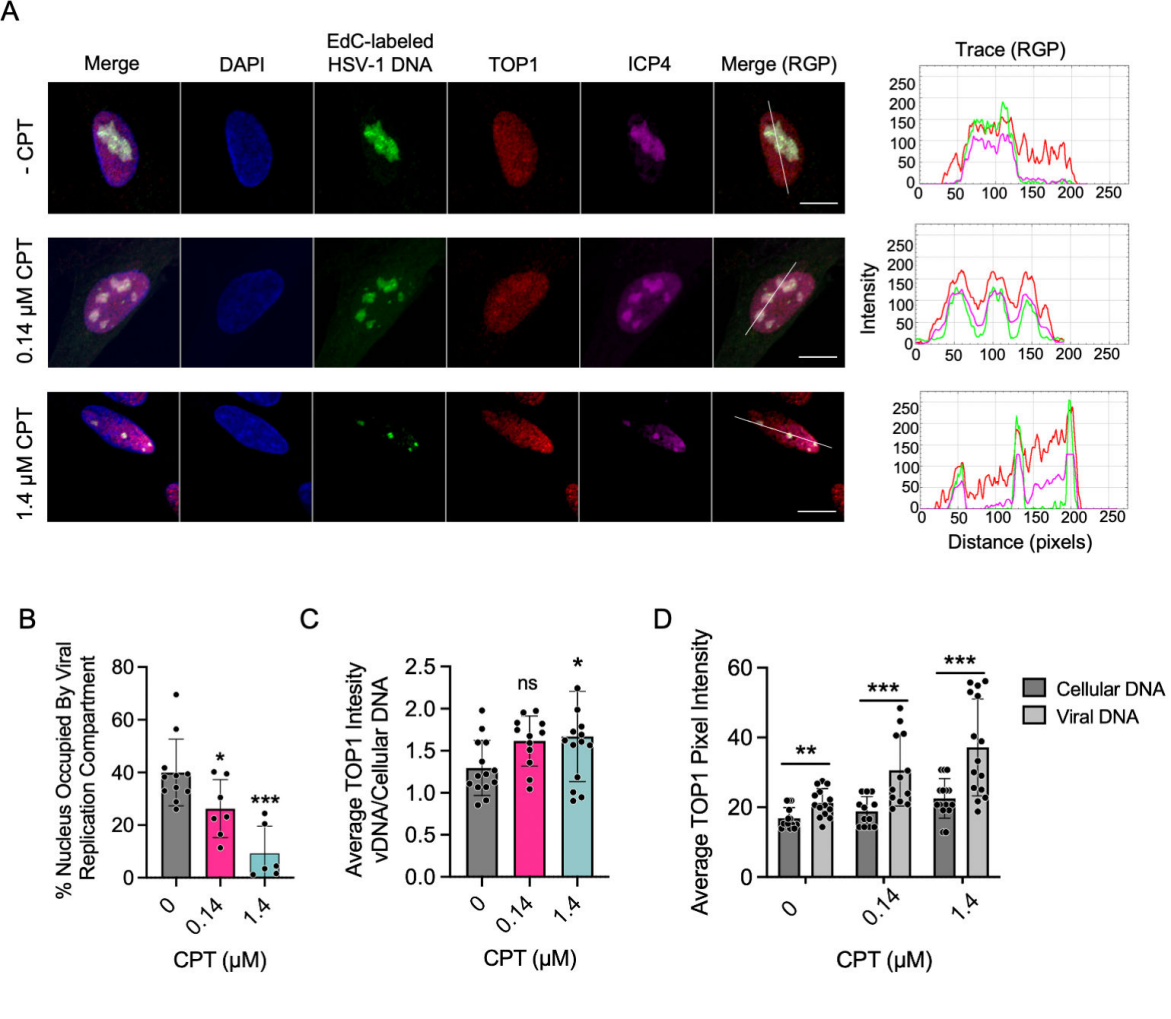
Inhibition with CPT enhances TOP1 colocalization with HSV-1 genomes and results in reduced replication compartment size. (**A**) TOP1 colocalizes with HSV-1 replication compartments in the presence of CPT. MRC-5 cells were treated with 0 (-CPT), 0.14, or 1.4 µM CPT and infected with KOS at an MOI of 10 PFU/cell. At 4 hpi, EdC was added to the growth medium, and at 6 hpi, the cells were fixed. Cellular DNA was stained with DAPI (blue), EdC-labeled viral DNA was tagged with Alexa Fluor 488 using click chemistry (green), and TOP1 (red) and ICP4 (purple) were visualized by indirect immunofluorescence. Merge displays all channels, and Merge (RGP) is the same, with DAPI omitted. Scale bars represent 10 µm. Trace files, generated in ImageJ, show the colocalization of viral DNA, TOP1, and ICP4 along the line drawn in the Merge (RGP) panel. (**B**) TOP1 inhibition with CPT reduces viral replication compartment size. The percentage of infected cell nuclei occupied by viral replication compartments at 6 hpi was determined in cells treated with 0 (*n* = 11), 0.14 (*n* = 7), or 1.4 (*n* = 6) µM CPT. The total area of each nucleus and replication compartment was quantified using ImageJ. Error bars represent standard deviations from the mean. *P*-values: * ≤0.05, ** ≤0.01, *** ≤0.001 as determined by one-way ANOVA followed by Dunnett’s multiple comparison test, comparing CPT-treated cells with the uninhibited control. (**C-D**) TOP1 pixel intensity is elevated in regions of viral DNA compared with cellular DNA. Average TOP1 pixel intensity was determined within regions of viral and cellular DNA using the ROI Manager in ImageJ. Data were generated from multiple images. In (**C**), the ratio of TOP1 intensity at regions of viral DNA (vDNA) compared to cellular DNA is presented. Error bars represent standard deviations. *P*-values: * ≤0.05, ** ≤0.01, *** ≤0.001 were determined by one-way ANOVA, followed by Dunnett’s multiple comparison test comparing 0.14 or 1.4 µM CPT conditions to an uninhibited control. In (**D**), the raw data used to generate the graph in (**C**) are presented with error bars representing standard deviations. Significance was determined by paired *t*-tests with pixel intensity of viral and cellular DNA paired within individual cells. *P*-values: * ≤0.05, ** ≤0.01, *** ≤0.001.

To investigate the effects of CPT treatment on TOP1 colocalization with HSV-1 DNA, the cells were inhibited with either 0, 0.14, or 1.4 µM CPT, infected with KOS, and replicating viral DNA was labeled with EdC from 4 to 6 hpi before fixation and staining at 6 hpi (Fig. 7A). Consistent with a DNA replication defect, CPT treatment resulted in a dose-dependent reduction in viral replication compartment size (Fig. 7B). In uninhibited cells, viral replication compartments occupy ~40% of the infected cell nucleus. When treated with 0.14 µM CPT, this decreased to ~25% and ~15% following 1.4 µM CPT treatment. In addition, CPT treatment resulted in an increase in TOP1 colocalization with viral DNA (Fig. 7C and D), suggesting that TOP1 is catalytically active on viral DNA and inhibition with CPT captures TOP1 on viral genomes. In all, CPT inhibition during infection results in a viral DNA replication defect, reduced replication compartment size, and increased TOP1 colocalization with viral DNA.

### TOP1 inhibition with CPT results in dose-dependent defects in viral gene expression

**Fig 8:**
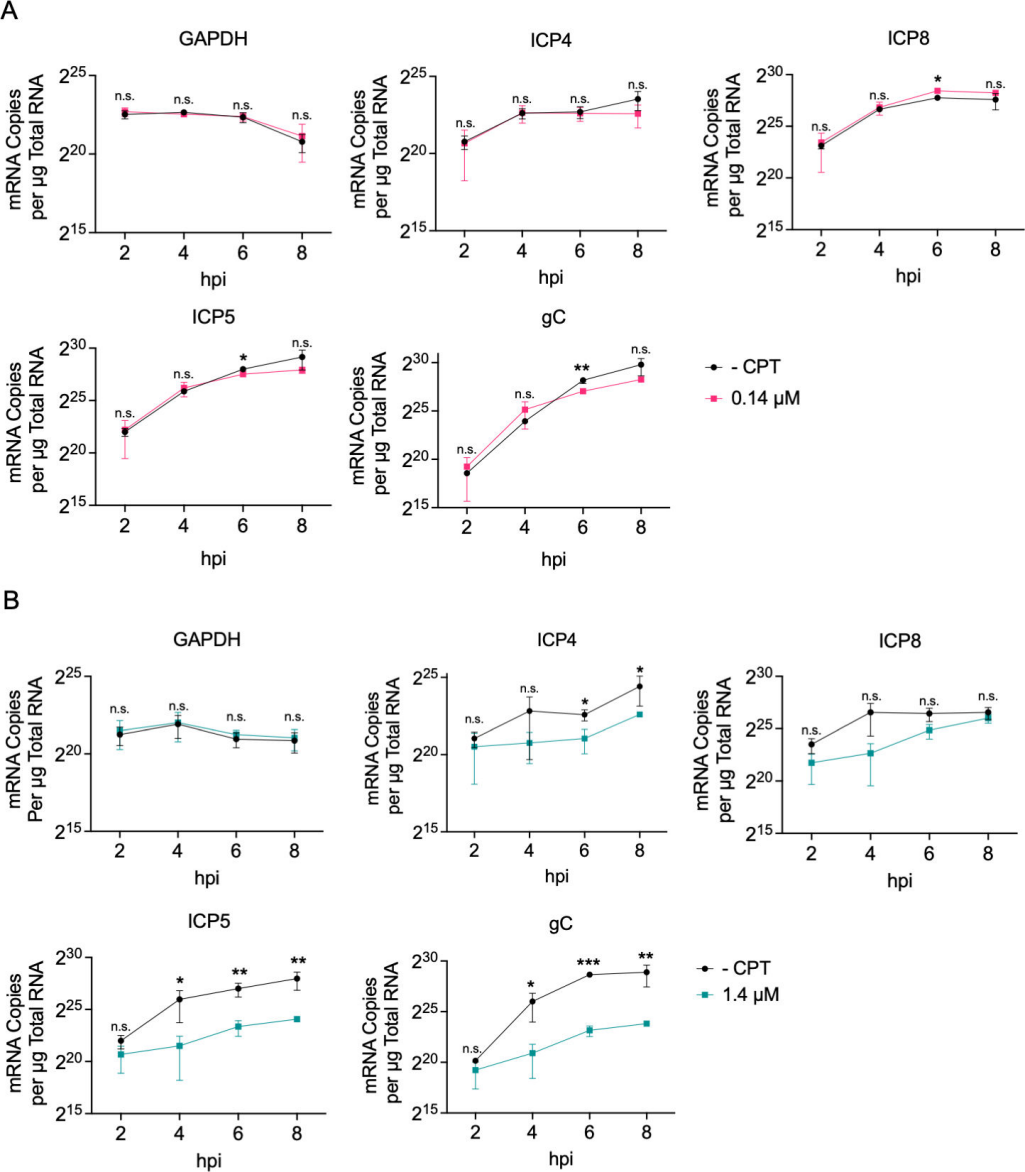
TOP1 inhibition by CPT results in defects in viral gene expression. MRC-5 cells were infected with strain KOS at an MOI of 10 PFU/cell in the presence or absence of 0.14 (**A**) or 1.4 (**B**) µM CPT. Samples were harvested at 2, 4, 6, and 8 hpi, followed by total RNA isolation. mRNA levels were quantified by RT-qPCR using primers specific for cellular GAPDH, as well as viral ICP4 (IE), ICP8 (E), ICP5 (LL), or gC (L) genes relative to a standard curve. Data points represent the average of three biological replicates. Error bars represent standard deviation comparing values for -CPT and +CPT conditions for each time point. *P*-values: * ≤0.05, ** ≤0.01, *** ≤0.001 as determined by unpaired Student’s *t*-tests. Statistical analyses were carried out on log10-transformed data.

Taken together, the data presented so far demonstrate that CPT causes a reversible and dose-dependent defect in HSV-1 DNA replication, resulting in a significant reduction in viral yield. To define the effects of TOP1 inhibition on HSV-1 gene expression, we quantified viral mRNA levels in the presence and absence of CPT. MRC-5 cells were infected in the presence of 0, 0.14, or 1.4 µM CPT, and total RNA was isolated at 2, 4, 6, and 8 hpi. These time points were chosen because they capture key events in the HSV-1 transcription cascade, including the onset and continued transcription of each viral gene class. RNA was reverse transcribed, followed by quantitative real-time PCR (RT-qPCR), and the relative quantities of representative mRNAs were determined by comparison to a standard curve of known quantities of viral or human DNA. Although statistically significant decreases in the levels of representative E (ICP8), LL (ICP5), and L (glycoprotein C (gC)) mRNAs were observed at 6 hpi when treated with 0.14 µM CPT, the general effects of CPT on viral mRNA levels were minimal at this concentration (Fig. 8A). In contrast, treatment of cells with 1.4 µM CPT resulted in a significant decrease in ICP4 (IE), ICP5 (LL), and gC (L) expression from 4 to 8 hpi, as well as an apparent decrease in ICP8 (E) expression at 4 hpi (Fig. 8B). ICP5 and gC had the most substantial reduction in mRNA levels. As a control, the levels of cellular GAPDH mRNA were also examined, and neither concentration of CPT influenced the expression of this gene. Overall, these data indicate that low concentrations of CPT have a modest effect on viral gene expression, whereas high concentrations result in a general defect in viral mRNA levels that is exaggerated for replication-dependent LL and L gene classes.

Collectively, these data suggest that at lower concentrations of CPT, the primary defect in infection stems from reduced viral DNA replication. In contrast, at higher concentrations, the decrease in viral yield likely results from combined defects in both viral gene expression and DNA replication.

## DISCUSSION

**Fig 9:**
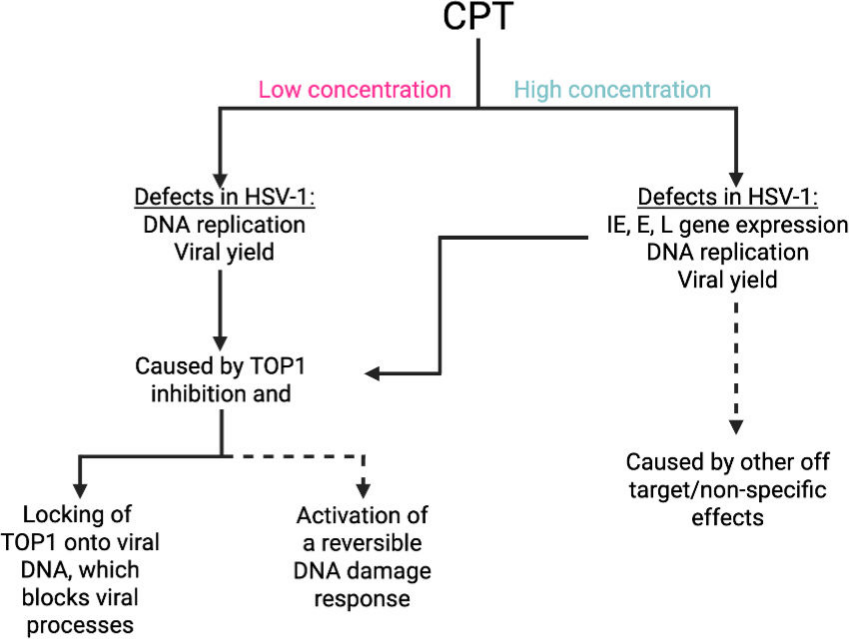
Summary of the effects of CPT treatment on HSV-1 infection. Treatment with CPT results in dose-dependent defects in HSV-1 infection. At a low concentration (0.14 µM), CPT treatment results in reduced HSV-1 DNA replication and viral yield. At this concentration, CPT inhibits infection by directly interacting with TOP1, likely either locking TOP1 on viral DNA or activating a reversible DNA damage response. At a high concentration (1.4 µM), CPT treatment results in reduced viral gene expression, DNA replication, and viral yield. At this concentration, defects are likely a result of both direct effects on TOP1 and indirect or non-specific effects of CPT treatment. Solid lines indicate direct support from data presented in this study, whereas dashed lines represent predictions based on observations from this study.

In this study, we investigated the role of TOP1 in HSV-1 infection and the inhibitory effects of CPT treatment. The first indication that TOP1 may play an active role in viral processes was the identification of TOP1 as a viral genome-associated protein by iPOND and related approaches (Fig. 1A) (29–31, 41). In addition, we and others have shown that TOP1 colocalizes with viral DNA during infection (Fig. 1B and C) (30, 32). Data presented here indicate that TOP1 inhibition with CPT results in reversible, dose- and MOI-dependent inhibition of HSV-1 infection, independent of cell type and strain (Fig. 2). Evidence that TOP1 is directly active on the viral genome includes observations that CPT cannot effectively inhibit viral infection after TOP1 knockdown (Fig. 4) and that there is an increased colocalization of TOP1 with viral DNA after CPT treatment (Fig. 7). However, TOP1 knockdown alone does not inhibit infection (Fig. 4), suggesting that TOP1 is not essential for viral processes and that its inhibition exerts a potentially dominant negative effect on infection. To analyze the effects of CPT treatment on consecutive steps in the infectious cycle, we examined viral gene expression and DNA replication in the presence of high and low concentrations of CPT. We also investigated how the timing of CPT addition affects viral yield. At 0.14 µM, CPT treatment results in a subtle transcription defect at 6 hpi (Fig. 8A) and a 5-fold to 7-fold reduction in viral DNA replication (Fig. 6), thus resulting in an approximately 10-fold reduction in viral yield (Fig. 2C and D), even when added after one or two rounds of viral DNA replication (Fig. 3A). At 1.4 µM, CPT treatment results in a reduction in mRNA expression for all viral genes tested (Fig. 8B) and a 500-fold to 1,000-fold reduction in viral DNA replication (Fig. 6). This results in an approximately 1,000-fold reduction in viral yield (Fig. 2C and D) and reduced viral replication compartment size (Fig. 7A and B), regardless of the time of addition (Fig. 3B). Taken together, the data presented here indicate that CPT is a potent inhibitor of HSV-1 infection. These results support the model that TOP1 associates with HSV-1 DNA and that topoisomerase activity plays a nonessential role in viral DNA replication and, to a lesser extent, viral gene expression (summarized in Fig. 9).

Topoisomerases have well-established functions in relieving topological stress during DNA replication and transcription. HSV-1 does not code for a topoisomerase, implying that a cellular enzyme may fulfill that role. Of the six cellular topoisomerases, TOP1, TOP2A, and TOP2B associate with viral DNA (29–32, 41). During DNA replication, the progression of replication forks introduces positive supercoiling in the template DNA, which is alleviated on cellular DNA by the activities of TOP1 and TOP2A (20, 44). Topoisomerase activity also contributes to the resolution of convergent replication forks and replication fork collisions (17). Transcription not only results in positive supercoils in front of the transcription bubble but also negative supercoiling of underwound DNA behind it (20, 23). These supercoils are generally relieved by TOP1 or TOP2B. Topoisomerases can also play regulatory roles in Pol II-mediated transcription. For example, TOP1 associates with the C-terminal domain of Pol II and is present but inactive at the sites of transcription initiation (23). During transcription elongation, TOP1 activity is stimulated by bromodomain-containing protein 4 (BRD4)-dependent phosphorylation of Pol II, resulting in release of the paused polymerase and transcription elongation. Collectively, cellular topoisomerases have the potential to play multiple roles in processes that occur on viral DNA. Based on the data presented here, TOP1 is likely active on HSV-1 genomes and contributes to viral gene expression and DNA replication.

In addition to the typical functions of topoisomerases, TOP1 can catalyze the removal of single ribonucleotides or stretches of RNA incorporated by DNA polymerases (20, 45). Ribonucleotides that are not removed by RNAse H2 are converted to single-strand breaks with 2´,3´-cyclophosphate 3´ ends by TOP1. These blocked ends are then removed by apurinic endonuclease, and this initiates an alternate pathway for the removal of ribonucleotides from DNA. TOP1 has also been implicated in the removal of ultraviolet-induced pyrimidine dimers in nucleotide excision repair-deficient cells (46) and may function in resolving Holliday junctions during DNA recombination (20, 47). Additionally, TOP1 can resolve RNA:DNA hybrids (R-loops) that form when RNA transcripts reanneal with the template strand of DNA (24). R-loops can be a source of transcription-transcription and transcription-replication conflicts, particularly at highly expressed genes. The HSV-1 genome contains multiple genes in proximity that are expressed in convergent, divergent, and overlapping manners. Therefore, TOP1 may help alleviate any R-loops that form. It is unknown whether TOP1 contributes to these processes during HSV-1 infection. However, our results provide a rationale for further investigation.

Although CPT is a potent inhibitor of viral processes, TOP1 knockdown does not affect viral yield (Fig. 4). This may seem contradictory but suggests that the accumulation of TOP1ccs is more obstructive than the absence of TOP1. The absence of TOP1 would result in reduced relief of supercoiling, leading to the gradual accumulation of topological stress. This stress could potentially be alleviated by other topoisomerases or compensated for by other processes, such as recombination. To investigate this possibility, we tested the effects of knocking down TOP1, TOP2A, and TOP2B in combination (Fig. 4) and found no reduction in viral yield. However, we did not investigate the potential compensatory roles that TOP3A or TOP3B could play, as these enzymes resolve DNA recombination intermediates (20).

It is also likely that TOP1ccs formed by CPT treatment physically obstruct DNA replication and transcription. The collision of replication forks and TOP1ccs may lead to the formation of DNA double-strand breaks and replication fork collapse, thus activating a DNA damage response that further inhibits infection (48, 49). Several pieces of evidence presented here shed light on the nature of a potential DNA damage response: (i) CPT is still effective at reducing viral yield when added at later times during infection (Fig. 3) when the host DNA damage response would already be dampened through the actions of the viral E3 ubiquitin ligase ICP0 (50). Therefore, a DNA damage response triggered by TOP1ccs may be attenuated compared to such a response in uninfected cells. (ii) The effects of CPT on viral yield are reversible (Fig. 2); therefore, any DNA damage response invoked by CPT must be reversible. Although we have not explored this possibility in more depth in this study, it is worth future investigation for potential antiviral approaches.

β-Lapachone, a catalytic TOP1 inhibitor mechanistically distinct from CPT (43), also suppresses HSV-1 infection at noncytotoxic concentrations (Fig. 5). Although we did not further investigate the specific steps in the infectious cycle that are blocked by β-Lapachone, recent work demonstrated that pretreatment of cells with β-Lapachone before viral infection completely inhibits the expression of the viral IE gene product, ICP4 (32). As ICP4 is a key regulator of viral transcription, this resulted in the shutdown of viral gene expression and DNA replication. Interestingly, this effect was not reversible when β-Lapachone was removed from 3 to 8 hpi, suggesting that there are key differences in the infection-inhibitory mechanisms of CPT and β-Lapachone. Like CPT, β-Lapachone could still inhibit viral processes when added at later stages of infection, consistent with TOP1 being active on viral DNA at all stages of infection, not just during IE gene expression. It is also important to note that β-Lapachone is less specific for TOP1 than CPT, as it can target TOP2 and can activate necroptosis and apoptosis (51–53). Considering these mechanistic differences, it is possible that inhibition of viral infection with β-Lapachone results from modulation of various other cellular pathways in addition to TOP1 inhibition.

Taken together, this study reveals mechanistic insight into the antiviral effects of CPT on HSV-1 infection. TOP1 has been implicated in the infection of several human herpesviruses, including HSV-2, Kaposi’s sarcoma-associated herpesvirus, and Epstein-Barr virus (34, 54, 55), suggesting that TOP1 is generally involved in processes that occur on herpes viral DNA and could be targeted for antiviral therapies. Although current antiviral treatments for HSV-1 can improve patient outlook, central nervous system infection and encephalitis can lead to poor patient prognosis (56–58). Additionally, antiviral resistance can arise during long-term treatment with antiviral drugs (59), highlighting the need to develop additional antivirals against lifelong HSV-1 infection. TOP1 is a chemotherapeutic target of CPT and its derivatives, as well as other drugs such as indolocarbazoles or indenoisquinolines (60, 61). The findings presented here propose the potential to target TOP1 or its interaction partners for the treatment of antiviral-resistant or deadly HSV-1 infections.

## MATERIALS AND METHODS

### Cells and viruses

MRC-5 (CCL-171), BJ-5ta (CRL-4001), and Vero (CCL-81) cells were obtained from and propagated as recommended by ATCC. MRC-5 cells were grown in Dulbecco’s Modified Eagle’s Medium (DMEM) supplemented with 10% fetal bovine serum (FBS). BJ-5ta cells were grown in DMEM supplemented with 10% FBS and 0.01 mg/mL hygromycin B. Vero cells were only used for plaque assays and were grown in DMEM supplemented with 5% FBS. HSV-1 strain KOS or 17syn + were used for infections as indicated in each experiment.

### TOP1 inhibition and HSV-1 infection

Unless otherwise indicated, CPT (MP Biomedicals 0215973225) or β-Lapachone (Cayman Chemical Company 15021) was added to the growth medium 1 h before infection. Infection was carried out in tris-buffered saline (TBS) at room temperature (RT) for 1 h, rocking every 10 min. Following adsorption, the cells were washed with warmed TBS, and the medium containing the appropriate concentration of CPT or β-Lapachone was added back to the cells. Infected cells were returned to the 37°C incubator, and the samples were harvested at the indicated times.

### Immunofluorescence imaging

MRC-5 cells were plated onto coverslips in 12-well dishes at a density of 1.67 × 10^5^ cells/well. Cells were either inhibited with CPT at the indicated concentration or left uninhibited and infected with an EdC-labeled KOS (KOS-EdC) or KOS at an MOI of 10 PFU/cell as described above. KOS-EdC was prepared as previously described (31). Cells were either incubated for 2 (KOS-EdC infected) or 6 (KOS infected) h before fixation. For the latter, at 4 hpi, the medium was replaced with DMEM supplemented with 25 µM EdC. Cells were fixed with 4% paraformaldehyde for 15 min at RT, and coverslips were stored in 1× phosphate-buffered saline (PBS) containing 3% bovine serum albumin (BSA) at 4°C overnight. All subsequent steps occurred at RT. Cells were permeabilized with 0.5% Triton X-100 in 1× PBS for 20 min, and then blocked with 1× PBS containing 3% BSA for 30 min. After blocking, Alexa Fluor 488 Azide (Invitrogen) was covalently attached to the EdC-labeled DNA via a click reaction for 30 min (62). Cells were stained with 600 nM DAPI for 30 min, followed by another 30 min incubation with a 1:200 dilution of rabbit α-TOP1 (Abcam AB109374) and a 1:200 dilution of mouse α-ICP4 (Abcam AB6514) antibodies. Cells were washed once with 1× PBS containing 1% BSA, and three times with 1× PBS. Alexa Fluor 594-conjugated goat α-rabbit IgG (Invitrogen A-11012) and Alexa Fluor 647-conjugated goat α-mouse IgG (Invitrogen A-21235) were added to the cells at a 1:200 dilution and incubated for 30 min. Coverslips were subject to a series of 3 washes with 1× PBS, mounted onto a slide using Shandon Immu-Mount, and imaged with a Nikon Eclipse Ti2 Inverted Confocal Microscope. ImageJ was used to measure viral replication compartment size. ImageJ RGB Profile Plot was used to generate traces. Average pixel intensity was measured using the ROI Manager in ImageJ.

### CellTiter-Glo luminescent cell viability assay

MRC-5 or BJ-5ta cells were plated at a density of 1.25 × 10^4^ cells/well in 96-well plates. Culture medium was removed and replaced with the fresh medium containing increasing concentrations of CPT or β-Lapachone. For controls, the wells were prepared to contain medium only with no cells or cells with medium containing 0.4% dimethyl sulfoxide (DMSO). Following incubation for 24 h at 37°C, cells were subjected to the CellTiter-Glo Luminescent Cell Viability Assay (Promega) as indicated in the manufacturer’s protocol. A SpectraMax iD3 plate reader was used to record luminescence. To account for any background signal, luminescence values from blank wells were averaged, and this value was subtracted from the luminescence reading for each condition. Percent viability was calculated by dividing the corrected luminescence of each condition by the corrected luminescence of the “cells-only” control and multiplying by 100.

### Viral yield assay

MRC-5 or BJ-5ta cells were plated in 6-well dishes at a density of 1 × 10^6^ cells/well. Infection was conducted as described above, using the indicated strain, inhibitor, and MOI for each experiment. At 24 hpi, cells and growth medium were collected by scraping. Cells were subject to three freeze-thaw cycles followed by sonication. Viral yield was determined by plaque assay in Vero cells.

### CPT removal assay

MRC-5 cells were plated in 12-well dishes at a density of 5 × 10^5^ cells/well. CPT inhibition (0.14 or 1.4 µM) and infection (MOI 10 PFU/cell) were carried out as indicated above. At either 3 or 6 hpi, the medium containing CPT was removed. Wells were washed with warmed TBS, and then, the medium without CPT was added. At 24 hpi, the virus was harvested and titered via plaque assay as indicated above.

### Time of CPT addition experiments

MRC-5 cells were plated at a density of 5 × 10^5^ cells/well in 6-well dishes and incubated overnight at 37°C. Cells were treated with 0.14 or 1.4 µM CPT at the indicated times either before or after infection. Infection and inhibition were carried out as described above. At 24 hpi, the virus was collected by scraping and titered via plaque assay.

### siRNA knockdown

Dharmacon SMARTpool siRNAs (Horizon - TOP1: L-005278-00-0005, TOP2A: L-004239-00-0005, TOP2B: L-004240-00-0005, Non-Targeting: D-001810-10-05) were resuspended and prepared according to the manufacturer’s protocol. Twelve-well dishes were pre-coated with 50 µL OptiMEM. siRNAs were prepared by combining and adding 2.5 µL RNAiMAX (ThermoFisher Scientific), 0.5 µL each siRNA (20 µM), and 127 µL OptiMEM (Gibco) to each well, followed by incubation at RT for 5 min. After incubation, the MRC-5 cells were plated onto the transfection mixtures at a sub-confluent density of 2 × 10^5^ cells/well in 1 mL DMEM containing 10% FBS. The medium was replaced at 24 h post-transfection, followed by HSV-1 infection or protein collection at 48 h post-transfection.

### Viral DNA replication assay

MRC-5 cells were plated at a density of 5 × 10^5^ cells/well in 6-well dishes. Following overnight incubation at 37°C, the cells were inhibited at either 0.14 or 1.4 µM CPT and infected at an MOI of 10 PFU/cell as described above. Total DNA was harvested at 2-h intervals from 0 to 12 and at 24 hpi in DNA extraction buffer (0.5% SDS, 400 µg/mL proteinase K, 100 mM NaCl). To measure the number of viral and cellular genomes, qPCR was performed. To quantify viral genomes, primers that recognize the TK gene were used. Quantification was based on a standard curve generated from known amounts of viral DNA. For cellular genome quantification, primers targeting GAPDH were used. Quantities were determined relative to a standard curve generated from known amounts of human DNA. Primer sequences are listed in Table 1 (63).

**TABLE 1:** Primer sequences used for qPCR

Gene	Forward primer (5′−3′)	Reverse primer (5′−3′)
ICP4	CGGTGATGAAGGAGCTGCTGTTGC	CTGATCACGCGGCTGCTGTACA
ICP8	CATCAGCTGCTCCACCTCGCG	GCAGTACGTGGACCAGGCGGT
ICP5	TGGATGGTATGGTCCAGATGC	GCACAACGGCGCTGCTCT
gC	GAGGAGGTCCTGACGAACATCACC	CCGGTGACAGAATACAACGGAGG
GAPDH	CAGAACATCATCCCTGCCTCTACT	GCCAGTGAGCTTCCCGTTCA

### Viral mRNA extraction and RT-qPCR

MRC-5 cells were plated at a density of 1 × 10^6^ cells/well in 6-well dishes and were incubated overnight at 37°C. The cells were inhibited and infected as indicated above, and RNA was isolated at 2, 4, 6, and 8 hpi using Trizol reagent (Invitrogen) according to the manufacturer’s protocol, including the DNA-free DNA Removal Kit (Invitrogen). As controls, uninhibited samples were collected at each time point. Samples were then subject to RT-qPCR as follows. RNA was reverse-transcribed using oligo(dT) primers (ThermoFisher Scientific). Reverse-transcribed cDNA was then used for qPCR to measure GAPDH, ICP4, ICP8, ICP5, and gC mRNA (Table 1) (63). Transcript copy numbers were calculated relative to standard curves generated from HSV-1 or Human DNA.

### Statistical analyses

All statistical analyses were carried out as indicated for each experiment using GraphPad Prism 10. Student *t*-tests were used to compare the difference between the means of two groups. ANOVA was used to compare the means of three or more groups. The following post hoc tests were used to determine the significance within ANOVA testing. Dunnett’s tests were used to compare each mean to that of the control, whereas Tukey’s tests were used when comparing each mean with every other mean.
